# Role of Tau protein in long COVID and potential therapeutic targets

**DOI:** 10.3389/fcimb.2023.1280600

**Published:** 2023-10-25

**Authors:** Bharat Marwaha

**Affiliations:** Department of Cardiology, Adena Health System, Chillicothe, OH, United States

**Keywords:** long covid, tau protein, small fiber neuropathy, chronic fatigue/myalgic encephalomyelitis, clinical symptoms of long covid

## Abstract

**Introduction:**

Long COVID is an emerging public health burden and has been defined as a syndrome with common symptoms of fatigue, shortness of breath, cognitive dysfunction, and others impacting day-to-day life, fluctuating or relapsing over, occurring for at least two months in patients with a history of probable or confirmed SARS CoV-2 infection; usually three months from the onset of illness and cannot be explained by an alternate diagnosis. The actual prevalence of long-term COVID-19 is unknown, but it is believed that more than 17 million patients in Europe may have suffered from it during pandemic.

**Pathophysiology:**

Currently, there is limited understanding of the pathophysiology of this syndrome, and multiple hypotheses have been proposed. Our literature review has shown studies reporting tau deposits in tissue samples of the brain from autopsies of COVID-19 patients compared to the control group, and the in-vitro human brain organoid model has shown aberrant phosphorylation of tau protein in response to SARS-CoV-2 infection. Tauopathies, a group of neurodegenerative disorders with the salient features of tau deposits, can manifest different symptoms based on the anatomical region of brain involvement and have been shown to affect the peripheral nervous system as well and explained even in rat model studies. Long COVID has more than 203 symptoms, with predominant symptoms of fatigue, dyspnea, and cognitive dysfunction, which tauopathy-induced CNS and peripheral nervous system dysfunction can explain. There have been no studies up till now to reveal the pathophysiology of long COVID. Based on our literature review, aberrant tau phosphorylation is a promising hypothesis that can be explored in future studies. Therapeutic approaches for tauopathies have multidimensional aspects, including targeting post-translational modifications, tau aggregation, and tau clearance through the autophagy process with the help of lysosomes, which can be potential targets for developing therapeutic interventions for the long COVID. In addition, future studies can attempt to find the tau proteins in CSF and use those as biomarkers for the long COVID.

## Introduction

The COVID-19 pandemic has changed the lives of millions across the globe. Recent statistics reveal that it has infected more than 760 million people and led to more than 6.9 million deaths worldwide. Initial research was focused on the acute complications of COVID-19, but now healthcare faces the significant burden of the long-term impact of COVID-19. The long-term complications of the disease include its detrimental impact on various organ systems based on the severity of the initial illness and underlying risk factors (Xie et al., 2022). Long COVID is a relatively new medical syndrome noticed among patients with a wide clinical spectrum. The WHO has described this entity as a constellation of symptoms ranging from systemic symptoms of fatigue, shortness of breath, cognitive dysfunction, and others lasting for at least two months, usually three months, from COVID-19 infection, with a relapsing and fluctuating course over time, which cannot be explained by an alternative diagnosis, irrespective of the severity of the initial illness or hospitalization status (https://www.who.int/emergencies/diseases/novel-coronavirus-2019/coronavirus-disease-answers?adgroupsurvey=%7Badgroupsurvey%7D&gclid=CjwKCAjwzruGBhBAEiwAUqMR8NuImw4LvO4C2n7n7MdNBo82efE8UcrchbGCeaEX4QVljInAFZfchoC6ycQAvD_BwE&query=Long+COVID&referrerPageUrl=https%3A%2F%2Fwww.who.int%2Femergencies%2Fdiseases%2Fnovel-coronavirus-2019%2Fcoronavirus-disease-answers-). CDC also recognizes this syndrome as a constellation of symptoms lasting more than 28 days (CDC, 2020).

## Clinical spectrum

Recently, a few studies have been conducted to understand the clinical spectrum of long COVID and its prevalence. In a prospective study on long COVID, 4182 incident cases of confirmed COVID-19 were reported on a study app. The majority of these respondents were from the U.K. (88.2%), followed by the U.S. (7.3%). The study reported that 13.3% (558) of the respondents had COVID-19 symptoms lasting for 28 days, with 4.5% (189) having experienced symptoms for 56 days and 2.6% (108) for a duration of more than 84 days. Fatigue was reported by 97.70%, followed by headache (91.20%), shortness of breath (70.80%), chest pain (60%), diarrhea (51%), loss of smell (72%), a persistent cough (68.20%), and delirium (30%) at the end of the 28-day time period (Sudre et al., 2021).

**Figure 1:**
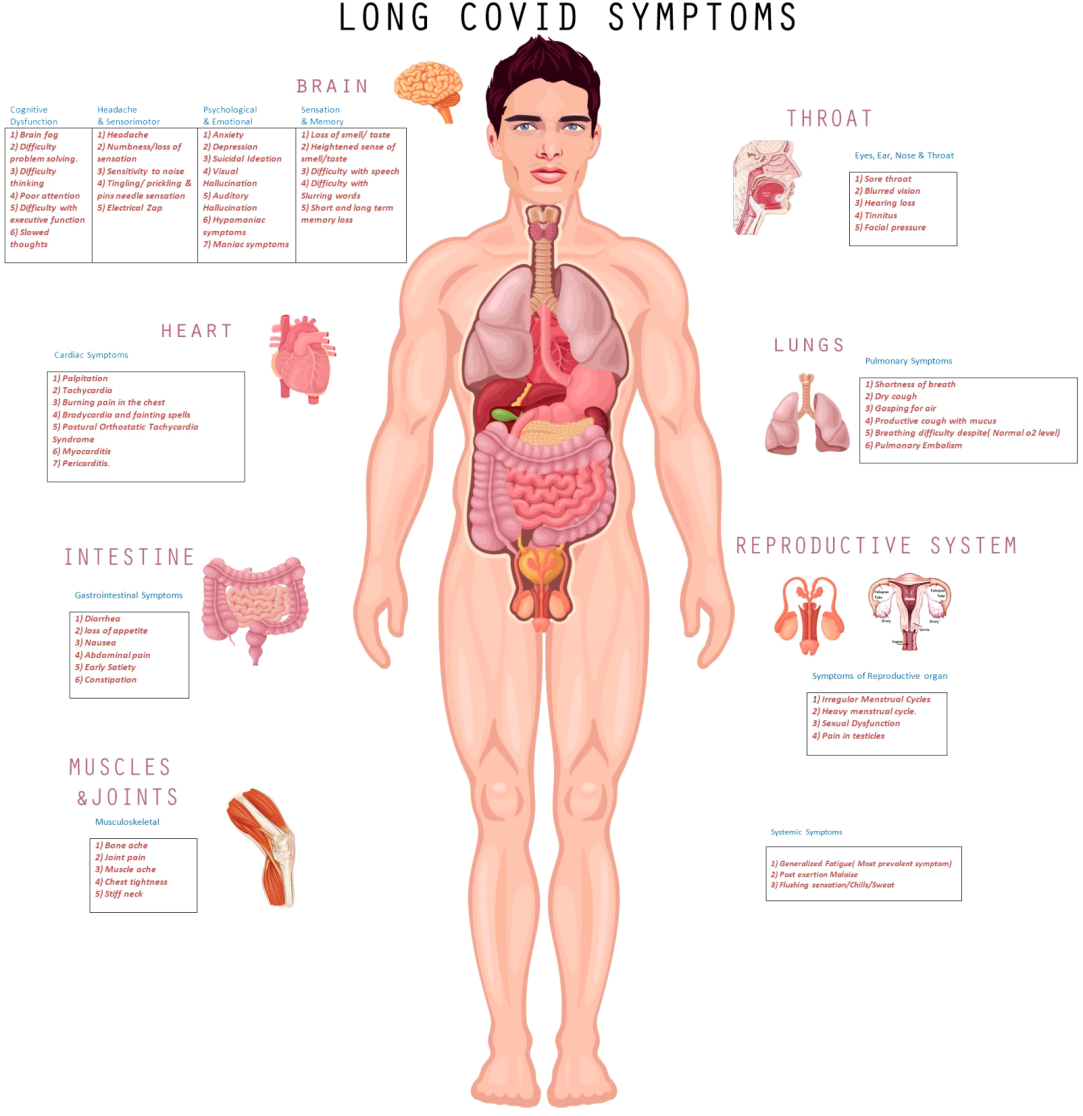
Pictorial Presentation of Clinical Manifestations of long COVID.

During the workup of 802 respondents, 27% were given a diagnosis of migraine, 14.7% myalgic encephalitis/chronic fatigue syndrome, 8.2% neuralgia, 19% POTS, and 8.6% myocarditis. The clinical spectrum of long COVID is shown in **Figure 1**, with a description of commonly manifested symptoms in various body-organ systems in corresponding tables.

In this cohort, 86%of the participants reported relapses, while 52.4% reported the relapse/worsening of symptoms in response to specific triggers, out of which 71% were related to physical activity, 58.9% to stress, 54% to exercise, and 46% reported during mental activity.

This study had some limitations, with respondents producing negative and absent test results, but it was conducted in the initial phase of the pandemic with limited availability of testing. The likelihood of false-negative test results was 20% after three days of the onset of symptoms, and up to 54% presented a false-negative initial RT-PCR (Arevalo-Rodriguez et al., 2020; Kucirka et al., 2020; Liu et al., 2020). This may have affected the accuracy of the testing.

In another study (Davis et al., 2021), 3762 participants with long COVID were followed up for seven months. The study attempted to elucidate the temporal trend of symptoms, triggers of relapses, and course of symptoms. The clinical spectrum of the symptoms in this group included systemic and post-exertional symptoms of fatigue (98%).

## Tau protein & COVID-19

Coronavirus belongs to the *Coronaviridae*family and the *Nidovirales* order. The first clinical case of COVID-19 was reported in Wuhan, China, on December 8th, 2019, with the initial genome sequence published on the virological website on January 10th, 2020. On February 11th, 2020, the International Committee on Taxonomy of Viruses named the novel coronavirus “SARS-CoV-2,” and the WHO named the disease “COVID-19” (King et al., 2012; Cui et al., 2019; Hu et al., 2021).ACE 2 is a receptor for SARS-CoV-2 infection (Hoffmann et al., 2020). Cardiac pericytes, blood vessels, respiratory epithelial cells, the kidneys, the intestines, the cortex of the human brain, the respiratory system, and the hypothalamus in the brain stem have these receptors (Hikmet et al., 2020; Lukiw et al., 2022). Systemic symptoms and multi-organ involvement in the acute phase are due to SARS-CoV-2 invasion via these receptors (Beyerstedt et al., 2021). SARS-CoV-2 infection can result in significant inflammatory and oxidative stress through dysregulated immuno-inflammatory pathways. Diffuse inflammatory markers were reported in >80% of COVID-19 patients’ brains, which could have contributed to the multiple neurological symptoms observed in a recently published study (Thakur et al., 2021). Hyposmia and hypogeusia frequently manifest in COVID-19. Hyposomia is reported in the initial stages of Alzheimer’s disease(AD), which expresses key pathological features of tau deposits.

In a recently published study (Reiken et al., 2022), the authors compared the autopsy tissue samples of COVID-19 brains to control samples. They have reported that oxidative stress and inflammatory pathways can trigger RyR(Ryanodine receptor) leakage. This can lead to dysregulated intracellular calcium levels, resulting in the activation of calcium-dependent enzymes and the hyper-phosphorylation of tau protein in COVID-19 brains compared to controls. This study evaluated the multiple aspects of pathogenesis, including oxidative stress, inflammatory markers, Ryanodine-receptor-two-complex oxidation, and the leakage and hyper-phosphorylation of tau protein. Further studies have reported that COVID-19 expresses oxidative-stress markers with a 3.8-fold increase in the glutathione disulfide (GSSG)/glutathione (GSH) ratio in the cerebral cortex compared to control brain-tissue samples.

Increased PKA and CaMKII activity, along with the phosphorylation/activation of AMPK and GSK3β, results in the hyper-phosphorylation of tau at multiple residues. COVID-19-brain lysates from aged patients showed increased tau phosphorylation at S199, S202, S214, S262, and S356. Younger patients showed tau phosphorylation at S214, S262, and S356 but not at S199 or S202, demonstrating increased tau phosphorylation and suggesting a tau pathology.

Stress-inflammatory/oxidative pathway activation results in increased transforming growth factor-β (TGF-β) signaling, which activates SMAD3 and increased NADPH Oxidase 2(NOX2) expression and activity at the RyR2 channel, leading to oxidation and calstabin 2 depletion from the channel macromolecular complex. This results in the destabilizing of the closed state and the inappropriate activation of channels at low cytosolic Ca2+ concentrations, resulting in pathological endoplasmic/sarcoplasmic reticular Ca2+ leakage. Increased RyR2-channel activity at rest was observed in COVID-19 channels compared to controls, suggesting the leaky biochemical feature of these channels.

This impaired calcium regulation can result in increased Ca2+/cAMP/PKA signaling, activating Ca2+-dependent enzymes and reduced neuronal firing.

This tau-phosphorylation model was tested in the *in vitro* model developed by Ramani et al. (2020) in their study on human-brain organoids derived from induced human pleura-potent stem cells. It was reported that the aberrant phosphorylation of tau protein leads to a series of events that can result in neuronal cell death. A SARS-CoV-2 sample taken from the patient was used for infecting the brain organoids, and respiratory epithelial cells were used as a control group in the study. Human-brain organoids of 15-day and 60-day age groups were studied after 2 and 4 days of initial infection (Ramani et al., 2020). The results showed that the virus preferably affects 60-day-old organoids with mature neuron cells rather than neuro-progenitor cells that are 15 days of age without any active signs of viral proliferation by replication and local inflammatory pathways as the potential mechanisms responsible for aberrant phosphorylation. Viral oxidative stress in neurons leads to the aberrant phosphorylation of p231T and the mislocalization in the soma of neuronal cells from axons (Ramani et al., 2020).

## Tau protein & immunopathology

Tau protein, in its physiological state, acts as a protective agent against peroxidation-induced DNA damage, regulates DNA packaging, and binds directly to its *in vitro* (Hua et al., 2003; Wei et al., 2008). Pathological tau in the disease state promotes filamentous actin, which can result in a rigid cytoskeleton and influence the dynamics of mitochondria, causing oxidative stress. Pathogenic tau can also cause the nuclear envelope to be involute, disrupting the nucleoskeleton. Pathogenic tau-induced oxidative stress and the disruption of lamin-rich nucleoskeleton activates silenced genes, leading to cell-cycle activation in neurons and triggering apoptotic cell death (Fulga et al., 2007; Arendt et al., 2010; Frost et al., 2014; Frost et al., 2016).SARS-CoV-2 infection can lead to hyper-inflammatory syndromes characterized by higher expressions of pro-inflammatory factors (Tay et al., 2020).

Immuno-pathological pathways with dysregulated innate immune responses have played an important role in the pathogenesis of COVID-19 (Vardhana and Wolchok, 2020). Higher levels of pro-inflammatory cytokines, including IL-1, IL-2, IL-4, IL-6, IL-7, IL-10, IL-13, IL-17, M-CSF, G-CSF, GM-CSF, and IP-10 have been reported in severe COVID-19 patients (Costela-Ruiz et al., 2020).The NOD-like receptor protein 3(NLRP-3) inflammasome is a key immuno-stimulator with multi-organ effects through dysfunctional immuno-inflammatory pathways in COVID-19 (Rodrigues et al., 2021; Dutta et al., 2022). NLRP 3 has also been shown in studies to influence the phosphorylation of tau protein and play an important role in tauopathies (Ising et al., 2019). Inflammasomes are integral components of innate immunity and play a role in regulating the inflammation response to cellular stress and infections (Mangan et al., [[NoYear]]).

Nuclear factor kappa-light-chain-enhancer of activated B cells (NF-κB) is another important inflammatory transcription factor that plays a pivotal role in regulating cellular response in neurodegenerative disorders, tauopathies, septic shock, viral infections, and chronic inflammatory response (Sun et al., 2022). There are five different transcription factors under this family that get activated in the presence of pro-inflammatory cytokines, leading to the translocation of P65/P50 dimers stored in the cytoplasm to the nucleus activating cognate κB motif,

resulting in the expression of the NF-κB target gene under the canonical pathway which has been extensively studied than noncanonical pathway activated by tumor necrosis factor (Motolani et al., 2021). NF-κB activated microglia, innate immune cells in the central nervous system, can accelerate the tau seeding and propagation of tau pathology, and inactivation of this transcription factor can lead to a slow down of this propagation demonstrated in the mice model (Wang et al., 2022). SARS-CoV 2 activates the NF-κB through multiple viral proteins, including nucleocapsid and spike protein, in proportion to viral load, and inhibition of this pathway contributes to better survival, although the exact mechanism is not fully elucidated yet (Davies et al., 2021; Gudowska-Sawczuk and Mroczko, 2022).

## Tau protein & tauopathies

In 1975, Weingarten et al. (1975a) found a protein contaminant along with microtubules, which is now known as the microtubule-associated protein tau or “tau,” which is critical for its stability. Subsequently, numerous studies reported that tau aggregates are primary pathological features of clinically diverse neurodegenerative diseases, which are termed “tauopathies.” These diseases include Alzheimer’s disease, progressive supra nuclear palsy, corticobasal syndrome, frontotemporal dementias, and chronic traumatic encephalopathy, defined by pathological tau-positive deposits in the brain (Weingarten et al., 1975b).

The symptoms of tauopathies are primarily based on the anatomical areas of the brain involved. Diagnosis is established through history, with ongoing efforts in translational research to establish the earlier diagnosis.Tau protein plays an important role in the stabilization of microtubules and converts 6S dimers of tubulin into the 36s rings required for microtubule polymerization, resulting in localization at axons of neurons under normal physiological conditions (Weingarten et al., 1975b). Multiple dynamic interactions of tau protein with tubulins regulate various aspects of neuronal growth and affect axonal sprouting, neurite polarity, neuroplasticity, and morphogenesis (Drubin et al., 1985; Liu et al., 1999; Takei et al., 2000).

Tau protein affects cell-cycle regulation by plasma-membrane interaction and tyrosine kinase (Brandt et al., 1995; Jensen et al., 1999), which plays a pivotal role in neuronal signaling and synaptic plasticity (Weingarten et al., 1975b; Hanger et al., 2019). Tau protein exhibits multiple features in disease states, which include aberrant phosphorylation, post-translation modifications, truncation, and aggregation into oligomers and larger insoluble filaments (Orr et al., 2017). Studies have reported evidence of the spread of pathogenic tau to neighboring cells, which can stimulate the pathogenic tau in these cells. Pathogenic tau can seed synaptically connected cells and lead to the stimulation and aggregation of natively folded tau in naive cells, leading to further progression of the disease (Frost et al., 2009; de Calignon et al., 2012). It may spread by extracellular micro-vesicles called “exosomes.” Tau-filled exosomes are present in cerebrospinal fluid and plasma of patients with mild Alzheimer’s disease and frontotemporal dementia (Goetzl et al., 2016).

A laboratory study conducted on transgenic mice expressing human non-mutant tau was used to assess the impact of the post-translational hyper-phosphorylation of tau on the central and peripheral nervous system after a period of 3 and 6 months (Marquez et al., 2021). The study’s findings revealed that aberrant hyperphosphorylation could lead to transient memory deficits along with peripheral neuropathy in the form of small-fiber and large-fiber neuropathy with tactile allodynia, altered thermal response, the slowing of motor-nerve-conduction velocity, and a reduction in intra-epidermal-nerve-fiber density (Marquez et al., 2021). Tauopathies have been reported among patients suffering from autonomic failure. Peripheral neuropathy can be secondary to disrupted cell signaling, loss of neurotrophic support, and structural alterations (Calcutt et al., 2008). More than 70% of peripheral neurons are small-fiber neurons (un-myelinated), and the estimation of intra-epidermal nerve-fiber density is the gold standard for the diagnosis of small-fiber neuropathy (Akihiko et al., 2021) Multiple ongoing research studies are underway in order to determine the role of tau protein as a potential biomarker to diagnose these disabling neuro-degenerative diseases at earlier stages and to compare it to imaging techniques.

PET imaging studies on Alzheimer’s disease have shown that cognition has a better correlation with tau pathology than with Aβ-plaque deposition (Brier et al., 2016; Johnson et al., 2016). Cerebrospinal fluid(CSF) phosphorylated (p-tau), total tau (T-tau), and amyloid-β 42 (Aβ42) are unique markers of Alzheimer’s disease. CSF-p-tau levels have shown a significant correlation during the evolution of the disease from preclinical to clinical dementia in A.D. compared to controls (Hansson et al., 2006; Shaw et al., 2009; Olsson et al., 2016) In a recent study published by Ashton et al. (2022), focusing on 171 participants, cross-sectional evaluation of CSF (p-tau 181, p-tau21, p-tau 231) and PET imaging showed that p-tau 231 is elevated in the preclinical stage and has potential for using it as biomarker and target for therapeutic interventions. Pathological tau can lead to cognitive dysfunction and small-fiber neuropathy and can result in a wide spectrum of clinical symptoms based on organ system involvement.

## Small-fiber neuropathy & long COVID

Small-fiber neuropathy is an umbrella term for neuropathies affecting thinly Aδ-myelinated and un-myelinated C-fibers. Various studies have reported a variable prevalence of disease, with an estimated incidence of 1.3/100,000, which increased over a period of time, and a prevalence of 13.3/100,000 (Brier et al., 2016). Small-fiber neuropathy can be mixed, purely sensory, or purely autonomic, depending on the involvement of the nerve fibers (Johnson et al., 2021).In a study conducted on 921 patients with pure small-fiber neuropathy, 53% had idiopathic small-fiber neuropathy; in the remaining patients, immunological diseases (sarcoidosis, Sjogren, celiac, and autoimmune) were reported in 19%and sodium-channel-gene mutations (SCN9A, SCN10A, and SCN11A) in 16.7%. Secondary causes, such as diabetes mellitus (7.7%), chemotherapy (2.2%), Vit B-12 deficiency (4.7%), alcohol abuse(3%), and monoclonal gammopathy of undetermined significance(1.4%),were reported in the study (Peters et al., 2013).

The National Institute of Health funded a study with the multicenter enrollment of patients without a prior history of neuropathy. The study included a total of 17 patients whose symptoms matched the definition of long COVID; after a follow-up of 1.4 years, it was revealed that 62.5% of the patients had a small-fiber-neuropathy diagnosis on lower-leg-skin biopsy (Oaklander et al., 2022),. Another study at the National Institute for Health and Care Excellence (NICE) in the U.K focused on 70 patients, including 30 controls and 40 patients with neurological symptoms four weeks post-COVID-19.The study identified small-nerve fiber loss in the latter group (Bitirgen et al., 2021),. In total, 85% of small-fiber-neuropathy patients have mild dysautonomia and neuropathy symptoms.

Another study, published by Nicholas Barizien et al. (2021), from France, with 39 participants without prior neurological complications from COVID-19 except for the loss of taste and smell, tested the hypothesis of dysautonomia among long COVID patients with fatigue. Heart-rate variability (HRV) with a change in position was used as a physiological marker for the evaluation of dysautonomia. Heart-rate variability has been used in the past to evaluate autonomic function. The HRV in Barizien et al.’s study was reflected by the NOL (nociception level) index assessed after changes in the positions of the patients and displayed in a range from 0–100. The NOL index is calculated by an artificial intelligence-driven algorithm based on multiple inputs from a noninvasive finger probe and has been validated for the assessment of the HRV in prior studies. The study showed a statistically significant difference in the NOL NOL index between a group of patients with long COVID and fatigue versus a healthy cohort with a P value 0.046.

In a study published in the *Journal of American College of Cardiology* on 41 patients reporting dyspnea post-COVID for 9 to 12 months, all the patients had normal pulmonary function tests, chest X-rays, echocardiogram, and chest computed tomography scans. Seven patients were offered invasive CPET; out of these, five patients had preload failure and met the criteria for myalgic encephalitis/chronic fatigue syndrome (Mancini et al., 2021),. Twenty-four patients (58.5%) had a predicted peak VO2 <80%, while 46% of the patients with dyspnea met the criteria for ME/CFS syndrome.

Phillip Joseph et al. (2021),performed a study to find the association between small-fiber neuropathy and myalgic encephalitis/chronic fatigue syndrome. The study enrolled 160 patients, 31% of whom had small-fiber neuropathy diagnosed by punch biopsy, and another control group featured 36 patients. The exclusion criteria were right atrial pressure (RAP) >6.5, resting or exercise pre- and post-capillary pulmonary hypertension, sub-maximal heart rate <80% predicted for age, minute ventilation to maximum voluntary ventilation >0.7 at anaerobic threshold, and a lack of skin biopsy for the diagnosis of small-fiber neuropathy. These participants were offered right-heart catheterization and cardio-pulmonary stress tests with incrementally increasing upright exercise. The study measured rest-to-peak changes in cardiac output (Qc) and systemic oxygen extraction by the Qc/Vo2 slope. The results were intriguing and showed impaired aerobic capacity, with the suggestion of two types of vascular dysregulation associated with small-fiber neuropathy as a presumptive etiology.

Decreased peak Vo2 and low cardiac output were reported in low-flow patients with decreased biventricular filling pressure secondary to low venous pressure that was not explained by intrinsic heart disease or pulmonary hypertension (Oldham et al., 2016). The degeneration of axons in small-fiber neuropathy can lead to impaired veno-constriction with the pooling of blood in the periphery, leading to low-flow preload failure. Similar abnormal venous pooling has also been observed among POTS patients as well (Streeten et al., 1988),. Decreased peak Vo2 can lead to the early onset of anaerobic metabolism and muscle fatigue.

The second group with low peak Vo2 had a high cardiac output and was found to have an impairment of systemic oxygen extraction. Immunohistochemical studies in the past have shown that small fibers regulate microvascular tone, primarily through sympathetic and parasympathetic cholinergic synapses on perivascular myocytes (Schuller et al., 2000). Dilated arterial-venous shunts have been noticed in histological studies among fibromyalgia patients who have considerable overlap with ME/CFS patients (Albrecht et al., 2013). These findings have been observed in the pathological examination of the microvasculature in chronic regional pain syndrome, which is a form of small-fiber neuropathy (Albrecht et al., 2006; Oaklander and Fields, 2009). These dilated shunts can lead to the bypass of oxygenated blood from capillaries and result in early anaerobic metabolism and muscle fatigue.

In the study, skin biopsy was used to establish the diagnosis of small-fiber neuropathy because of its high specificity, 91%, but it has lower sensitivity, 58% (Fabry et al., 2020). Small-fiber malfunction is not identified by the biopsy, but it can contribute to this dysregulation, although this contribution is very difficult to quantify (Duchesne et al., 2018).Further studies have suggested that small-fiber neuropathy can lead to impaired aerobic metabolism, resulting in symptoms of fatigue, which is also the most prevalent symptom of long COVID. In one of the largest studies on long-COVID patients, 14% of the patients had a diagnosis of ME/CFS during the workup on long COVID. De-conditioning has been frequently considered as a confounding factor in chronic-fatigue patients. De-conditioning is usually associated with low cardiac output, with impaired ventricular compliance with elevated filling pressure, contrary to the low biventricular pressure and high cardiac output noticed in the above study (Saltin et al., 1968; Stickland et al., 2006; Keller et al., 2014).

## Potential treatment options and targets for therapeutic interventions

Tau protein undergoes post-translational modifications, which can reduce its binding to microtubules, leading to an increased level of cytoplasmic tau proteins. These tau aggregates can assemble, leading to the formation of oligomers, straight filaments, and paired helical filaments, which are neurotoxic. Paired helical filaments and straight filaments contribute to the formation of NFTs, a hallmark of neurodegenerative disease (Wang et al., 2021).

Therapeutic approaches for tauopathies have multidimensional aspects, including targeting the post-translational modifications, tau aggregation, and tau clearance through the autophagy process with the help of lysosomes. Tau-protein clearance is also enhanced by vaccines or antibodies (Soeda and Takashima, 2020).

Phosphorylation is a key post-translational modification that can be influenced by protein kinases and phosphatases. Phosphoprotein phosphatase(PP2A) is a key phosphatase, accounting for over 70% of tau dephosphorylation, in contrast to the multiple protein kinases involved in the phosphorylation of tau protein (Ferrer et al., 2005; Liu et al., 2005). Although the targeting of one enzyme because of its substrate specificity and several regulatory subunits is promising, it has been challenging to target this enzyme to develop therapeutics (Wolfe, 2016),. Various clinical trials have tested protein kinases for developing therapeutic agents. Glycogen synthase kinase (G.S.K. 3β) has been associated with phosphorylation at 26 sites of tau protein, and its activity level correlates with the progression of the neurodegenerative process, and the over-activation of this enzyme contributes to tau hyper-phosphorylation (Yamaguchi et al., 1996; Guo et al., 2017). So far, the clinical trials targeting this enzyme have not been successful in reversing the disease process.

Recently, multiple newer agents targeting tau aggregation have undergone trials with the potential to improve clinical outcomes. When tested in a mouse model, rosmarinic acid(RA), a polyphenol found in Lamiaceae herbs, showed that it inhibits the accumulation of phosphorylated tau protein and improves spatial memory (Yamamoto et al., 2021). Resveratrol, a non-flavonoid polyphenol-rich in red wine and grape skin, induces the dephosphorylation of tau by interfering with the MID1–PP2A complex and the down-regulation of protein kinase for tau phosphorylation glycogen synthase kinase(G.S.K. 3β) signaling pathways (Xia et al., 2010; Jhang et al., 2017; Schweiger et al., 2017). The drug treatment showed improvement in cognitive function in a mouse model, but rapid metabolism in the liver and intestines leads to poor bioavailability, and analogs have been developed to improve the bio availability (Chimento et al., 2019; Sun et al., 2019).

Curcumin, a primary component of the Indian spice turmeric, has been shown to have an inhibitory effect on tau aggregation with a reduction in tau oligomer (Rane et al., 2017). The poor bioabsorption of curcumin and rapid degradation likely led to no therapeutic benefits in trials (Vareed et al., 2008). Analogs with better bio-absorption have undergone clinical trials (Chen et al., 2018). Folic acid has been shown to inhibit tau aggregation by stabilizing the tau in the native state and can reduce tau phosphorylation by regulating PP2A methylation (Li et al., 2015; Ghasemzadeh and Riazi, 2020). Tau degradation involves both the ubiquitin–proteasome system and the autophagy–lysosome system (Lee et al., 2013).

Tau-protein clearance is increased by treatment with vaccines or antibodies, and multiple clinical trials for tauopathies are currently testing this approach (Troquier et al., 2012; Ando et al., 2014; Sandusky-Beltran and Sigurdsson, 2020). AADvac1, a synthetic peptide consisting of amino acids 294–305 of the tau, generates antibodies against the tau protein. It has been shown to reduce tau pathology in an animal model (Kontsekova et al., 2014), and it has been tested in a clinical trial(ADAMANT;NCT02579252) in phase 1 and phase 2 on Alzheimer’s patients. It has been shown to lead to decreased cognitive decline among younger patients, albeit without improvements across all age groups, and will undergo a phase-3 clinical trial.ACI-35 immunotherapy has been used to target S-396/404 tau phosphorylation by generating specific antibodies, showing reduced soluble and insoluble tau in the brain and improving survival in animal-model studies (Theunis et al., 2013). ACL -35 has been redesigned to generate as ACL-35.030 to generate a more robust response (Soeda and Takashima, 2020).

A multicenter phase-1b/2a study is being conducted to evaluate the safety and immunogenicity of this vaccine in Alzheimer’s disease (AD) patients(NCT04445831).BIIB092 is a humanized monoclonal antibody developed against an N-terminal fragment of tau (extracellular tau) secreted from familiar AD-patient-derived pluripotent stem cells. It is undergoing a phase-2 clinical trial for AD (TANG0; NCT03352557) (Soeda and Takashima, 2020). Oligonucleotide therapy targeting antisense and Si-RNA aims to control the onset and progression of the disease by regulating the protein expression levels. In transgenic mice, tau antisense oligonucleotides (AS0) reduced the amount of tau mRNA by 50% and showed inhibition of neuronal loss, hippocampal atrophy, and neuronal loss (DeVos et al., 2017). The A.S.O. drug, BIIB080, is in a phase-1/2 double-blind, placebo-controlled trial(NCT03186989) on mild AD patients.

## Discussion

Post-COVID complications pose a significant public health burden, with a recent study from CDC reporting its prevalence at between 20 and 25% among COVID-19 patients (Bull-Otterson et al., [[NoYear]]).

The extensive literature review above revealed the tau deposits in the brain by examining the tissue samples from autopsies of COVID-19 patients compared to controls. Another *in-vitro* model on human-brain organoids infected with the COVID-19 virus showed the aberrant phosphorylation of the tau protein, p tau 231, resulting in neuronal cell death after infection with SARS-CoV-2.A variety of body stresses, including viral infection, can trigger pathological tau protein, which can possibly spread to healthier naive cells through synapses, resulting in functional and structural changes in the peripheral and autonomic nervous system, as discussed above. This can result in peripheral, autonomic, and small-fiber neuropathy, as revealed in transgenic-rat-model studies, and can manifest in the form of a diverse range of clinical symptoms.

Long-COVID patients have predominant symptoms of fatigue, post-exertional malaise, cognitive dysfunction, peripheral neuropathy, and autonomic dysfunction, which can potentially be explained by tauopathy neuropathy, including small-fiber neuropathy, as discussed above.

*Limitations*: In the literature review, there is no direct study available to elucidate the pathogenesis of long COVID or the direct impact of tauopathy on long COVID. Our literature review has explored the autopsy studies showing aberrant tau phosphorylation in COVID-19 patients and *in vitro* studies showing similar findings of aberrant phosphorylation resulting in neuronal cell death. Multiple immuno-histopathological studies have shown the NLRP 3 inflammasome’s key role in COVID-19 as well as its role in the tauopathies along with NF-κB, a transcriptor factor’s direct impact on COVID-19 and tauopathies.

Apart from the above-mentioned pathophysiology of long COVID, there may be other possible pathways that can potentially explain the Long COVID, which needs to be explored to address this emerging disease.

## Conclusion

I propose a prospective clinical study for the evaluation of the role of tauopathy-induced neuropathy among long-COVID-19 patients with fatigue. The further evaluation and testing of CSF for abnormally elevated tau protein in human organoids infected with COVID-19 could be the first step in the development of a biomarker for the diagnosis of long COVID. Elucidating the underlying mechanism of aberrant phosphorylation can help us to develop new targets for therapeutic interventions for long COVID and other tauopathies in the future.

## Author contributions

BM: Writing – original draft.
